# Avoidance versus use of neuromuscular blocking agents for optimizing video laryngoscopy‐assisted tracheal intubation: A protocol for a systematic review with meta‐analysis and trial sequential analysis

**DOI:** 10.1111/aas.70008

**Published:** 2025-02-20

**Authors:** Andreas Creutzburg, Anders Kehlet Nørskov, Michelle Icka Christensen, Arash Afshari, Lars Hyldborg Lundstrøm, Louise Bill, Helene Korvenius Nedergaard, Matias Vested

**Affiliations:** ^1^ Department of Anaesthesiology, Operation and Trauma Center Center of Head and Orthopaedics, Rigshospitalet Copenhagen Denmark; ^2^ Department of Anaesthesiology Nordsjællands Hospital Hillerød Denmark; ^3^ Collaboration for Evidence‐based Practice and Research in Anaesthesia (CEPRA) Copenhagen Denmark; ^4^ Department of Clinical Medicine University of Copenhagen Copenhagen Denmark; ^5^ Department of Anaesthesiology and Operation Juliane Marie Centre, Rigshospitalet Copenhagen Denmark; ^6^ Department of Anaesthesiology Sygehus Lillebælt Vejle Denmark; ^7^ Department of Anaesthesiology Sygehus Lillebælt Kolding Denmark

**Keywords:** neuromuscular blocking agents, tracheal intubation, video laryngoscopy

## Abstract

**Background:**

Both the European Society of Anaesthesiology and Intensive Care and the Difficult Airway Society recommend the use of neuromuscular blocking agents (NMBAs) to facilitate tracheal intubation and to reduce the risk of complications. Even though it is recommended to use video laryngoscopy for intubation, especially in circumstances where difficult tracheal intubation is expected, the evidence for the combination of video laryngoscopy and NMBAs is sparse. This protocol outlines a systematic review of the effect of avoidance versus use of NMBAs for tracheal intubation during video laryngoscopy in adults.

**Methods:**

This protocol is made in accordance with reporting items for systematic reviews and meta‐analyses protocols recommendations. We intent to include randomised controlled trials assessing the effect of avoidance versus use of NMBAs during video laryngoscopy to facilitate tracheal intubation (either nasal or oral) in the adult population. The primary outcome is failed first‐pass intubation. Our secondary outcomes include adverse and serious adverse events. A thorough search will be done to identify relevant trials, including CENTRAL, MEDLINE, EMBASE, BIOSIS, Web of Science and CIHNAL. Furthermore, trial registries will be searched for unpublished trials. Each trial will be evaluated for bias. We will use appropriate packages in R to perform the meta‐analysis. Additionally, we will perform trial sequential analysis on the meta‐analysis of our primary outcome. Lastly, we will employ the GRADE approach and create “Summary of Findings” tables.

## 1 INTRODUCTION

Tracheal intubation during anaesthesia may be associated with complications such as sore throat, hoarseness, laryngeal haematoma, and pharyngeal contusion.^1^, ^2^, ^3^ Major complications when tracheal intubation is difficult include death, hypoxic brain damage, aspiration of gastric contents, and pneumonia.^4^, ^5^

The European Society of Anaesthesiology and Intensive Care (ESAIC) and the Difficult Airway Society (DAS) recommend the use of neuromuscular blocking agents (NMBAs) to optimise tracheal intubation conditions by paralysing the laryngeal muscles^6^, ^7^ and increase the chance of successful first attempt, especially when tracheal intubation is difficult. Also, the use of a video laryngoscope is recommended in case of difficult tracheal intubation (DTI).^8^, ^9^

However, NMBAs are associated with serious side effects, such as intraoperative awareness^10^ and anaphylaxis^11^ along with an increased risk of pulmonary complications,^12^ and a need for reintubation due to the residual effect of neuromuscular blockade after extubation.^13^, ^14^ As an alternative, a short‐acting synthetic opioid^15^, ^16^, ^17^, ^18^ can be administered. However, such opioids may increase the risk of hypotension and bradycardia.^17^, ^19^

Evidence addressing the possible effects of avoidance versus use of NMBA during video laryngoscopy on tracheal intubation conditions is sparse.^20^, ^21^

This protocol outlines a systematic review of the available evidence to assess the effect of avoidance versus use of NMBA to facilitate tracheal intubation in adults using a video laryngoscope.

## 2 OBJECTIVE

With this systematic review, we aim to evaluate the effects of avoidance versus use of NMBAs on intubation conditions in adults during video laryngoscopy.

### 2.1 Methods

This protocol is developed in accordance with the PRISMA‐P statement. The final review will be reported following the PRISMA guideline,^22^ and the methodology will follow the recommendations outlined in the *Cochrane Handbook for Systematic Reviews and Interventions* (the Cochrane Handbook).^23^ The strength of the evidence will be evaluated by the GRADE approach.^24^

The protocol for this systematic review will be registered on PROSPERO before data extraction begins.

## 3 ELIGIBILITY CRITERIA

### 3.1 Types of studies

We will include randomised controlled trials (RCTs) with no language or letter restriction. Since video laryngoscopes of interest were introduced after 2000, only trials commenced after 2000 will be eligible. We will include unpublished clinical trials, but only if trial data and methodological descriptions can be obtained in written form through direct contact with the authors.

We will include cluster‐randomised trials and trials with more than two intervention groups. We will exclude trials using quasi‐randomisation designs and observational trials.

### 3.2 Types of participants

We will include adult patients (≥18 years) requiring in‐hospital tracheal intubation, including both oro‐tracheal and naso‐tracheal intubation, using a video laryngoscope of any clinical relevance for general anaesthesia for both elective and acute procedures.

If trials have overlapping age groups or are not restricted to only video laryngoscopes, the trial will be considered for inclusion after full‐text screening. Corresponding authors will be contacted to obtain data regarding relevant patients.

### 3.3 Types of intervention and control

Intervention: Avoidance of NMBAs to facilitate tracheal intubation (e.g., placebo, other drugs than NMBAs, no drugs) during video laryngoscopy in any form, dose, and duration.

Control: Use of NMBAs to facilitate tracheal intubation during video laryngoscopy in any form, dose, and duration.

The use of NMBA is defined as the control group since it is recommended to optimize tracheal intubation condition.^6^, ^7^, ^25^

### 3.4 Types of outcome measures

#### 3.4.1 Primary outcomes

1. Incidence of failed first‐pass intubation: This is defined as failed tracheal tube placement after the first attempt to introduce the video laryngoscope into the patient's mouth, assessed dichotomously.

#### 3.4.2 Secondary outcomes

1. Incidence of failed intubation: Defined as the inability to perform tracheal intubation, assessed dichotomously.
2. Incidence of serious adverse events (SAEs): Defined by the individual trials including any untoward medical occurrence (e.g., anaphylaxis, pulmonary aspiration, oesophageal intubation, hypoxia, hypotension, arrhythmia, re‐intubation, respiratory failure, awareness) assessed dichotomously. If the authors have not defined any SAEs, we evaluate the incidence of death, life‐threatening conditions, persisting disability, or which results in or prolongation of hospitalisation as defined by the International Council for Harmonisation of Technical Requirements for Registration of Pharmaceuticals for Human Use—Good Clinical Practice (ICH‐GCP).^26^
3. Incidence of adverse events (AEs): One or more events of upper airway discomfort or injury recorded (e.g., sore throat, hoarseness, vocal cord lesion, minor pharyngeal injury, dental injury).
4. Intubation condition: Defined by the individual trials, assessed dichotomously as either clinically acceptable or unacceptable.

### 3.5 Search methods for identification of trials

#### 3.5.1 Electronic search

We will search the following databases with the help of a Cochrane‐trained information specialist:

- Cochrane Central Register of Controlled Trials (CENTRAL) in the Cochrane Library (latest issue).
- MEDLINE (2000 to current date).
- EMBASE (2000 to current date) if access is available to the review team.
- BIOSIS (2000 to current date).
- International Web of Science (2000 to current date).
- Cumulative Index to Nursing and Allied Health Literature (CINAHL).

The systematic search strategy will be customised for MEDLINE and adapted for the other databases. See Appendix A for the search strategy for the MEDLINE search strategy.

We will use a systematic and sensitive search strategy to identify relevant RCTs. An additional search will be done before submission to reveal any newly published trials relevant to this systematic review.

#### 3.5.2 Searching other sources

We will search for unpublished trials on:

- https://clinicaltrials.gov/.
- The World Health Organisation International Clinical Trials Registry Platform (ICTRP) Portal.
- The ISRCTN registry (www.isrctn.com).
- The Australian New Zealand Clinical Trials Registry (http://www.anztr.org.au).
- European Union Drug Regulating Authorities Clinical Trials Databases (EudraCT).
- Clinical Trials Information System (CTIS).

We will hand‐search the reference lists of relevant, included trials and systematic reviews for potentially eligible trials.

### 3.6 Data collection and synthesis

#### 3.6.1 Trial selection

All reports found through the search strategy will be managed using the Covidence software.^27^ Duplicates of the same trial will be merged before screening for title and abstract. Two independent reviewers (AC and MIC) will screen the titles and abstracts to find eligible trials and remove irrelevant trials. If the two reviewers find duplicates of the same trial, these will be marked, and the primary trial will be included for full‐text screening. This process will follow the guide given in section 4.6.2 of the Cochrane handbook.^23^ Disagreement between the two reviewers will be resolved by discussion. If continued, a third reviewer will resolve the issue. We will pilot‐test our selection process to validate the process before commencing screening of potentially eligible trials. We will document the selection of trials in a flow chart as recommended in the PRISMA statement.^22^ Excluded trials and the primary reason for exclusion will be summarised in the “Characteristics of excluded trials” tables.

#### 3.6.2 Data extraction and management

The two authors (AC and MIC) will independently extract and collect data in a standardised form in Covidence. The data extraction form will be tested before the beginning of data extraction. They will not be blinded to the author, institution, or the publication source of the trial. Disagreement will be resolved as described above. If necessary, we will contact the corresponding author of the included trials for further information regarding this review's outcome measures and the risk of bias components.

In some trials, patients may be randomised to several intervention groups, as seen in dose‐finding trials. We will include and combine all relevant comparator or intervention groups into a single comparator or intervention group, as recommended in the Cochrane Handbook.^23^

#### 3.6.3 Data items

We will extract the following information:

- General information: Corresponding author, title, year of publication, language, country.
- Trial characteristics: trial design, number of sites, duration of enrolment, details of random sequence generation, allocation sequence concealment, number of randomised participants, number who received the intended intervention, number lost to follow‐up/withdrew consent, number analysed, methods used to prevent and address missing data, source of funding, conflict of interest.
- Participants: Age, sex, setting, inclusion and exclusion criteria, weight, height, BMI, comorbidities, ASA classification, type of procedure, type of anaesthesia, intubation method, method for monitoring depth of neuromuscular block, type of video laryngoscope, type of video laryngoscope blade.
- Intervention: Definition of the intervention group, anaesthetics (hypnotics, opioids, local anaesthetics) (type, dose, timing, administration), contaminant drugs (e.g., vasoactive drugs).
- Control: Definition of the control group, NMBA (type, dose, timing, administration), anaesthetics (hypnotics, opioids, local anaesthetics) (type, dose, timing, administration), contaminant drugs.
- Outcomes: As specified under outcomes.
- Additional information: DTI predictors reported by the authors in the individual trials (e.g., Mallampati score, thyromental distance, neck movement, mouth opening/incisor gab, ability to mandible subluxation), time from induction to start of tracheal intubation.

We will summarise the characteristics of all trials that will contribute to each comparison and present these in the “Characteristics of included studies” table.

#### 3.6.4 Assessment of risk of bias in included trials

To judge the overall risk of bias for each outcome, we intend to use the classic risk of bias tool from 2011 as recommended by Cochrane in section 8.5.^28^ Two reviewers (AC and MIC) will independently assess the risk of bias for each outcome. If there are discrepancies in the judgement and an inability to reach a consensus, a third reviewer will be consulted. For all domains, we will use the signalling questions with the following options: yes, probably yes, probably no, no, or no information as described in Chapter 8 of the Cochrane Handbook.^23^ Subsequently, the reviewers (AC and MIC) will reach a risk of bias judgement and assign one of the three levels to each domain: “low risk of bias”, “unclear risk of bias”, and “high risk of bias”.^23^ We will assess the following domains:

- Bias due to the randomisation process (selection bias) including random sequence generation and allocation concealment.
- Bias due to participants and personnel (performance bias).
- Bias due to missing outcome data (attrition bias).
- Bias due to outcome assessor (reporting bias).
- Bias due to selection of the reported results (selection bias).
- Other types of bias include bias arising from the source of funding and conflicts of interest.

The overall risk of biased judgement will be summarised for each domain across all included trials listed in this protocol. Only if all domains are judged to be of low risk of bias, the trial will be judged as “low risk of bias”. If we judge at least one domain as unclear but no high risk of bias domain, the overall risk of bias judgment will be “unclear risk of bias”. A trial will be judged as “high risk of bias” if at least one domain is judged to be at high risk of bias or “some concerns” for more than one domain. Only in case of lack of information in one or more of the key domains of bias and there is no clear indication that the study is at “high risk of bias”, we will apply the overall assessment “no information” as recommended in Chapter 8 of the Cochrane Handbook.^23^ We will present the results from the risk of bias assessment in a “risk of bias graph” and a “risk of bias summary figure”.

If 10 or more trials are included in the meta‐analysis, we will create funnel plots to assess for publication bias or “small study effects” and apply an Arcsine‐Thompsen test.^29^

### 3.7 Dealing with missing data

We will contact the corresponding author in case of missing trial data. Where standard deviations or other necessary data cannot be obtained, we will calculate them using other data from the trial if appropriate, as outlined in Chapter 6 of the Cochrane Handbook.^23^ We will not perform imputation in any of the analyses.

One reason for missing reported outcomes may occur when non‐significant results are selectively withheld from publication.^23^ Therefore, we intend to explore selective outcome reporting by comparing publications with their protocols, whenever the latter are available. If only protocols are available, we intend to investigate why publication was never done by the trial investigators.

We will perform a modified intention‐to‐treat (mITT) analysis including all randomised participants who undergo video laryngoscopy‐assisted tracheal intubation if possible. As no consensus exists on how missing data should be handled, different approaches may be appropriate in different situations.^30^ In case of missing data, we will use a “complete‐case analysis” for our primary outcome, which simply excludes all participants for whom outcome data were missing from the analysis.

### 3.8 Data synthesis

We will use Rstudio^31^ with the appropriate meta‐packages to perform the meta‐analysis. We will report dichotomous outcomes as risk difference (RD) and relative risks (RR)with 95% confidence intervals (CIs). We will only report RR if RR and RD results are similar. We expect mortality to be a rare event; therefore, we will calculate the Peto odds ratio (OR) as outlined in Chapter 10 of the Cochrane Handbook.^23^ Additionally, we will report the standardised mean difference (SMD) with 95% CIs for continuous outcomes. We will use guidelines set out in Chapter 6 of the Cochrane Handbook to synthesise data.^23^ If included trials use different scales, we will ensure that higher scores are consistent in direction.

If data are not reported in a format that can be entered directly into a meta‐analysis, we will assess the data and convert it to the required format if appropriate using the information in Chapter 6 of the Cochrane Handbook.^23^

Meta‐analysis will be undertaken where outcome measures are comparable between at least two included trials. Further, meta‐analysis will be performed if heterogeneity measures (not higher than moderate heterogeneity) indicate that pooling of results is appropriate. If outcome data are unsuitable for meta‐analysis, we will aim to summarize effect estimates by following the guidance in Chapters 9 and 12 of the Cochrane Handbook.^23^

As a supplement to the planned meta‐analysis, we plan to perform Trial Sequential Analysis (TSA). We will do so to prevent a potential increase in the risk of type I error due to multiple testing.^32^ Moreover, it will provide important information in the context of the level of evidence. Additionally, TSA provides important information regarding the need for future trials and required information size.^32^ We will apply trial sequential monitoring boundaries (TSMB) according to an information size suggested by the trials with low risk of bias and an a priori 30% relative risk reduction (RRR) of the primary outcome. As mortality is expected to be of low incidence,^5^ we will also perform TSA with an information size estimated based on an a priori 50% RRR of mortality.

In some trials, patients may be randomised to multiple intervention and control groups, or both, as seen in dose‐finding trials. We will combine all relevant experimental intervention groups of the trials into a single intervention group and combine all relevant control groups, as described in Chapter 6 of the Cochrane Handbook.^23^

### 3.9 Assessment of heterogeneity

We will assess statistical heterogeneity by quantifying results using diversity (*D* ^2^)^33^ and inconsistency factor (*I* ^2^) statistics.^34^ *p* ≤ 0.10 will be seen as an indicator of significant heterogeneity. Specific thresholds for the interpretation of *I* ^2^ statistics can be misleading when few trials are included,^23^ but we will define thresholds for the I^2^ as follows:

- Low degree of heterogeneity: <25%.
- Moderate degree of heterogeneity: 25%–50%.
- High degree of heterogeneity: >50%.

We plan to undertake both a fixed‐effects model and a random‐effects model. In case of *I* ^2^ = 0, we will only report the results from the fixed‐effects model. However, if *I* ^2^ >50%, indicating substantial heterogeneity, we emphasize the results from random‐effects models. Currently, we believe that the risk of substantial heterogeneity is possible due to the broad inclusion of different types of patient types, concurrent medication, and outcome reporting.^23^

### 3.10 Additional analyses

#### 3.10.1 Subgroup analysis and investigation of heterogeneity

The following subgroup analyses will be performed on the primary outcome:

1. Use of NMBA versus avoidance of NMBA, comparing low risk of bias with high or uncertain risk of bias.
2. Use of NMBA versus avoidance of NMBA, comparing depolarising with non‐depolarising NMBA.
3. Use of NMBA versus avoidance of NMBA, comparing remifentanil with other analgetic regimens.
4. Use of NMBA versus avoidance of NMBA comparing trials using anticipated difficult airway as an inclusion criterium with those that did not use anticipated difficult airway as an inclusion criterium.

We will use the Chi^2^ test to test for subgroup interaction as described in Chapter 10 of the Cochrane Handbook.^23^ We consider <0.05 to be indicative of significant interaction between opioids without NMBA effect on DTI and the subgroup category.

#### 3.10.2 Sensitivity analyses

We plan to perform the following sensitivity analyses:

- Trials with “low risk of bias” versus trials with “some concern” or “high risk of bias”.
- Trials without missing data for the primary outcome versus trials with missing data on the primary outcome.

### 3.11 Confidence in cumulative evidence

We will use the principles of the GRADE system to assess the quality of the body of evidence associated with specific outcomes. Here we will encompass the five GRADE key considerations of risk of bias, inconsistency, indirectness, impression, and publication bias. We will construct a “Summary of findings” table using the GRADEpro GDT software.^35^ This will be done for the following outcomes:

- Difficult tracheal intubation.
- All‐cause mortality.
- One or more events of upper airway discomfort or injury (e.g., sore throat, hoarseness, vocal cord injuries, minor pharyngeal injuries).
- One or more major, serious events (defined as gastric aspiration, brain, or cardiac injury).

We will use methods and recommendations as outlined in Chapter 14 of the Cochrane Handbook.^23^ We will justify all decisions to downgrade or upgrade the certainty of evidence using footnotes. Two reviewers (AC and MIC) will independently make judgments about the certainty of the evidence, with disagreements resolved by discussion or involving a third reviewer.

## 4 DISCUSSION

The impact of avoidance versus use of NMBAs to facilitate tracheal intubation conditions using video laryngoscopy is still not well understood since most data are based on direct laryngoscopy. This protocol aims to evaluate the effect of NMBA use on intubation conditions during video laryngoscopy.

We will pool data from randomised controlled trials that utilise video laryngoscopes of any kind, as current guidelines do not differentiate, but all recommend the use of video laryngoscopes for DTI.^7^, ^36^

We have chosen the incidence of failed first‐pass intubation as the primary outcome because, at this point, no international consensus exists on the definition of DTI.^4^, ^37^ Failed first‐pass intubation is a known risk of serious adverse events such as death and hypoxia‐induced brain damage.^4^, ^38^ Because no consensus on the definition of DTI is established, dichotomizing outcomes based on scale‐derived cut‐offs is controversial. However, we find that a frequency outcome will be tangible for clinicians.^39^ Moreover, it allows us to combine more trials, which may be beneficial when aggregating data.

Since we investigate perioperative interventions with little or no long‐term systemic effect, we expect most SAEs to appear shortly after attempted tracheal intubation. We will include registered SAEs regardless of the postoperative follow‐up period.

In addition, we plan to perform TSA to investigate the needed information size to conduct a trial with a low risk of bias to be able to detect the incidence of difficult tracheal intubation.

Overall, we believe that this systematic review will provide important information regarding optimizing tracheal intubation using a video laryngoscope with or without NMBAs.

## AUTHOR CONTRIBUTIONS

Matias Vested is the guarantor. Andreas Creutzburg drafted the manuscript. Michelle Icka Christensen, Matias Vested, Anders Kehlet Nørskov, Lars Hyldborg Lundstrøm, Louise Bill, Helene Korvenius Nedergaard and Arash Afshari contributed to the development of the conceptualisation, introduction, eligibility criteria, selection of outcomes, data extraction criteria, the risk of bias assessment strategy and data analysis strategy. Andreas Creutzburg developed the search strategy. All authors reviewed the manuscript, provided feedback, and approved the final version of this protocol.

## FUNDING INFORMATION

No funder, sponsor, or institution has had any influence on the process of writing this protocol or the final review.

## CONFLICT OF INTEREST STATEMENT

Andreas Creutzburg, Michelle Icka Christensen, Anders Kehlet Nørskov, Lars Hyldborg Lundstrøm, Helene Korvenius Nedergaard, Matias Vested, and Louise Bill declare no conflict of interest. Arash Afshari is a co‐author of the latest ESIAC guideline on peri‐operative management of neuromuscular blockade.^6^ Arash Afshari has no financial conflict of interest.

## Data Availability

The data that support the findings in this systematic review and meta‐analysis will be available from the corresponding author upon reasonable request.

## APPENDIX A

### MEDLINE SEARCH STRATEGIES

Search strategy is reported according to PRISMA‐Search (PRISMA‐S).^41^

Ovid MEDLINE(R) ALL <1946 to May 20, 2024>

**Table:**

1.	exp Neuromuscular Blocking Agents/	25410
2.	(dihydrochandonium or fazadinium or metocurine or suxamethonium or suxamethon or succinylcholine or rapacuronium or mivacurium or mivacron or atracurium or doxacurium or vecuronium or pancuronium or tubocurarine or gallamine or pipecuronium or rocuronium or esmeron or esmerone or zemuron or cisatracurium or nimb+ex or nimbex or anectine or quelicin or celocurine or lysthenon or (neuromuscular adj3 agent*) or neuromuscular block* or muscle relax* or NMBA*).ab,kf,ti,tw.	41413
3.	1 or 2	51141
4.	Laryngoscopy/	14270
5.	Intubation, Intratracheal/	40399
6.	(intubat* or endotracheal tube or laryngoscop*).ab,kf,ti,tw.	88934
7.	(glidescope* or mcgrath* or c‐mac* or truview* or pentax* or "airway scope" or airtraq* or secmac* or king vision* or A P Advance or AWS* or EndoSTROB or Storz* or VLP*).ab,ti,kf,tw.	14074
8.	"video?laryngoscop*".ab,ti,kf,tw.	1692
9.	4 or 5 or 6 or 7 or 8	122465
10.	randomized controlled trial.pt.	613546
11.	controlled clinical trial.pt.	95537
12.	randomized.ab,ti.	703720
13.	randomi?ed.ab,ti.	836585
14.	placebo.ab,ti.	255217
15.	drug therapy.sh.	31171
16.	randomly.ab,ti.	434745
17.	trial.ab,ti.	808357
18.	groups.ab,ti.	2708597
19.	10 or 11 or 12 or 13 or 14 or 15 or 16 or 17 or 18	3978892
20.	exp animals/ not humans/	5223076
21.	19 not 20	3455601
22.	3 and 9 and 21	2689
23.	limit 22 to yr="2000‐Current"	1498
